# Enhancing Care Transitions for Older People through Interprofessional Simulation: A Mixed Method Evaluation

**DOI:** 10.5334/ijic.3055

**Published:** 2017-11-13

**Authors:** Susie Sykes, Lesley Baillie, Beth Thomas, Judy Scotter, Fiona Martin

**Affiliations:** 1 School of Health and Social Care, London South Bank University, 103 Borough Road, London, SE1 0AA, GB; 2 School of Health, Wellbeing and Social Care, Open University, Walton Hall, Milton Keynes, MK7 6AA, GB; 3 Simulation and Interactive Learning (SaIL) Centre, Guy’s and St Thomas’ NHS Foundation Trust, GB; 4 Clever Together, 2 Primrose Street, London, EC2A 2EX, GB

**Keywords:** older people, care transitions, integrated care, collaboration, interprofessional working, simulation

## Abstract

**Introduction:**

The educational needs of the health and social care workforce for delivering effective integrated care are important. This paper reports on the development, pilot and evaluation of an interprofessional simulation course, which aimed to support integrated care models for care transitions for older people from hospital to home.

**Theory and methods:**

The course development was informed by a literature review and a scoping exercise with the health and social care workforce. The course ran six times and was attended by health and social care professionals from hospital and community (n = 49). The evaluation aimed to elicit staff perceptions of their learning about care transfers of older people and to explore application of learning into practice and perceived outcomes. The study used a sequential mixed method design with questionnaires completed pre (n = 44) and post (n = 47) course and interviews (n = 9) 2–5 months later.

**Results:**

Participants evaluated interprofessional simulation as a successful strategy. Post-course, participants identified learning points and at the interviews, similar themes with examples of application in practice were: Understanding individual needs and empathy; Communicating with patients and families; Interprofessional working; Working across settings to achieve effective care transitions.

**Conclusions and discussion:**

An interprofessional simulation course successfully brought together health and social care professionals across settings to develop integrated care skills and improve care transitions for older people with complex needs from hospital to home.

## Introduction

Older people often have complex needs and thus require services from health and social care professionals from various organizations and sectors [1,2], with a resulting risk of duplication of services and inconsistencies in approach [2]. Integrated care could therefore particularly benefit older people with complex needs, especially as they experience regular transitions between services [3,4,5,6]. There are a range of definitions of integrated care but Goodwin [7] argued for a person-centred perspective, with integrated care being viewed as ‘an overarching term for a broad and multi-component set of ideas and principles that seek to better co-ordinate care around people’s needs’. Stein [8] identified that everyone involved in delivering integrated care needs to attain further competencies, including both technical and behavioural competencies that go beyond those traditionally taught and which require social and emotional intelligence, but that few integrated care initiatives have invested in the education that staff need to deliver integrated, people-centred care. The project presented in this paper focused on the pilot of an interprofessional simulation course as an educational intervention to support integrated care initiatives for improving care transitions for older people with complex needs from hospital to home.

Care transitions are complex and multidimensional and there are a range of facilitators and barriers to success [9]. Transition types include transfers from home-to-hospital, hospital-to-home, hospital-to-skilled care facility, and skilled care facility to-home and/or homecare [10]. Transitional care has been defined as a set of actions ensuring the coordination and continuity of healthcare as patients transfer through different settings and different levels of care within the same setting [11,12] The quality of care related to transitions is important [12], particularly as people are more vulnerable to health risks during transitions [9]. High-quality transitional care is particularly necessary for older adults with multiple chronic conditions and complex care needs and their family carers [13]. However, care transitions continue to be a problematic area of policy and practice within England [5,14,15] and internationally [10,16]. Recently, Orvik et al. [2] argued that transitional care has become of central importance in efforts to improve the quality of health services and patient safety globally. Initiatives to improve transitional care should focus on the people involved and how they can effectively collaborate. There needs to be high quality communication within the interprofessional team and with the family for successful care transitions to home for older people [17] and effective collaborative practice is essential to prevent adverse events related to transitions [2].

Miller [18] asserted that collaboration between health and social care services is vital for providing integrated care but that those working in health can find it difficult to understand the nature of ‘social care’. Miller [18] further highlighted that there can be negative attitudes of health care professionals towards social care professionals and vice versa, resulting in barriers to collaborative working practices. Interactions between professionals with different backgrounds require mutual respect and trust [19] as well as high quality communication skills [20]. Actual or perceived boundaries between staff in different settings, and a lack of communication between hospital and community staff, can be problematic and may hinder integrated care in practice and adversely affect care transitions [15]. Planning for transitions from hospital to home is most effective when started at the time of hospital admission during assessment and involves effective interprofessional teamwork and partnership working with patients and their families [21,22,23]. Previous studies have indicated that healthcare professionals lack awareness of transition requirements and the needs and available services for older people following transfer home [24,25]. An educational programme on discharge planning for Japanese nurses was found to have a sustained effect on their attitudes and knowledge [26], although application of learning to practice was not investigated.

The current study took place in south London, where Southwark and Lambeth Integrated Care (SLIC) was developed as a virtual integration, which is defined as being a commitment to work collaboratively without the organisational change of a horizontal or vertical integration [27]. Lambeth and Southwark are London boroughs with a complex and diverse population of about 600,000 people [28] and SLIC aimed to promote integrated care across the populations of these two boroughs, by bringing together general practices and community care, two National Health Service (NHS) Trusts that provide acute hospital care, a mental health trust, social care providers and health and social care commissioning groups. One of SLIC’s workstreams focused on care transitions of older people with complex needs from hospital to home, with one objective being to address the associated educational needs of health and social care professionals across settings. Graham et al. [29] studied the transitional care needs of vulnerable older people in the US and identified five levels to be considered: (1) the individual; (2) the interpersonal; (3) the organisational; (4) the community environment; and (5) policy. This project was aimed at addressing levels 1) and 2) and supporting integrated care at an individual and interpersonal level, with an interprofessional simulation course as an educational intervention.

This paper reports on the development, piloting and evaluation of an interprofessional simulation course for health and social care professionals from varied settings, who were involved in care transfers of older people in south London. Interprofessional education can prepare individuals to engage in meaningful collaboration [30,31,32], thus supporting a ‘collaborative practice-ready’ workforce that can better respond to local health needs [33]. The course was run as a pilot and aimed to support SLIC’s integrated care models through promoting best practice for care transfers of older people and providing opportunities for developing skills for integrated care. The course development was informed by a review of the literature and a scoping exercise to ensure it was based on existing evidence as well as local need. The literature review used an integrative approach and systematically searched key databases for current literature reporting best practice on care transfers of older people from hospital to home. The scoping exercise elicited the local health and social care workforce’s perceived educational needs, regarding care transitions from hospital to home for older people with complex needs. This paper includes the scoping exercise method and results, followed by a summary of the interprofessional simulation course development and its implementation as a pilot. The findings from the mixed method evaluation of the course are then presented. The study aims were to:

1. Identify staff perceptions of their learning about care transfers of older people following an interprofessional simulation course;
2. Explore whether and how staff applied their learning from the course into their practice and the perceived outcomes.

## Methods

The methods presented provide a summary of the approach taken in the scoping exercise that informed the course development, the course design and implementation, and the study design used to evaluate the course.

### Scoping exercise

The scoping exercise, conducted July–September 2014, included a range of sources accessed through various activities. Initially consultation meetings took place with key individuals in a range of hospital and community services, a social care team and a community multidisciplinary team (see Table 1). Each meeting took an informal conversational approach and lasted about 45 minutes, with notes taken and written up fully afterwards. Areas discussed were: what works well in care transfers from hospital to home for older people with complex needs; what works less well and needs improving; what are the training needs of staff involved; and how could training be delivered most effectively. The consultation process was iterative, with issues identified in earlier meetings raised for discussion during later meetings. To include patient perspectives, the project team reviewed results from SLIC’s survey of local patients over 65 years (n = 94) who had been discharged home from one of the two main hospitals in 2013. The literature review findings around discharge planning, patient, family and carer involvements as well as multi disciplinary team involvement were integrated with themes from the consultation meeting summaries and patient survey results, leading to a preliminary list of training needs, which were reviewed with staff (n = 23: nursing, medical, allied health and social work) on older people’s wards at the two main hospitals. Finally, observation of multi-disciplinary team (MDT) meetings on three older people’s wards, where care transfers were being discussed, took place. These meetings further highlighted the complexity of planning care transfers for older people returning home, the importance of knowing the patients and their home and family situation, and the need for effective interprofessional teams and integrated working across settings. The observation notes were reviewed against the training needs and confirmed that the proposed training content was comprehensive.

**Table 1:** Consultation meeting participants.

Role and service
Social Care team (n = 9 team members)
Hospital Matrons for older people’s wards (one from each acute hospital: n = 2)
Community team leads (n = 3)
Discharge Coordinator Managers (one from each acute hospital: n = 2)
Social work manager
Lead occupational therapist
Lead physiotherapist
Community multi-disciplinary team (occupational therapists, physiotherapists, nurses) (n = 12 team members)

The scoping exercise identified training needs within six key themes of: patient and family involvement, interprofessional working, integrated working, communication and documentation, assessment and the discharge process. Many staff considered that there was some progress in improving care transfer processes for older people with complex needs locally and most identified various current and recent SLIC projects that were having a positive impact. As regards delivery of training, there was strong support for simulation, interprofessional learning and the use of complex patient scenarios. Staff also identified that training opportunities that enabled them to learn more about each other’s roles, and service provision and resources across hospital and community, would be beneficial.

### Course Design

The aim of the course was to promote best practice for person-centred care transfers of older people with complex needs from hospital to home, with identified objectives being to:

1. Draw upon shared experience and knowledge to identify and promote best practice for safe transfer of care from hospital to home;
2. Develop and enhance participants’ care transition skills, including: effective communication, assessment and evaluation of individual patient needs, and the ability to work in an integrated way within a multi-agency, multi professional arena.

The course was interprofessional and comprised mixed-modality simulation activities including immersive and life-simulations, which reflected what staff do in their everyday practice within hospital and community environments. Simulation is defined as a method ‘to replace or amplify real experiences with guided experiences, often immersive in nature, that evoke or replicate substantial aspects of the real world in a fully interactive fashion’ [34]. Simulation-based education has been shown to increase patient safety and improve clinical and patient management skills [35,36,37]. The course closely reflected the themes identified in the scoping exercise and was designed to reflect a single patient’s journey from hospital to home with community care. The simulation scenarios were based on real life situations that local people described and were designed to reflect best practice and give participants the chance to learn in a safe and realistic environment. Professional actors served as simulated patients and family members during the scenarios. The participants also experienced the challenges older people with complex needs may experience when performing everyday activities, through wearing a suit that replicates physical constraints i.e. reduced movement, vision and hearing. Deliberate practice, reflection and feedback are the educational processes that underpin simulation [38], all of which were built into the course.

The course was interprofessional and all courses included participants from a range of settings, both hospital and community. Interprofessional education (IPE) is defined as occurring when ‘students from two or more professions learn about, from and with each other to enable effective collaboration and improve health outcomes’ [33] and provides opportunities for different professions to learn how to work effectively together [39,40]. Simulation-enhanced IPE (sim-IPE) approaches have been increasingly developed as a way of providing interprofessional collaboration experiences in clinical and community settings [41]. Sim-IPE occurs ‘when participants and facilitators from two or more professions are engaged in a simulated healthcare experience to achieve shared outcomes’ [37, p. 293]. Interprofessional simulation has been found to increase understanding of the roles of other professionals [42,43], improve attitudes towards other professions [44] and improve interprofessional communication [45,46,47]. Interprofessional simulation courses that focused on care transitions were found to improve understanding of professional roles [48] and the care team’s attitudes [49].

All course delegates were provided with pre-reading about teamwork and communication and also material specifically about care transitions. The course ran six times between June and September, 2015. The first course was reviewed upon completion and minimal changes were made for subsequent courses. In total, 49 staff attended and the participants were of varied seniority and professional backgrounds: social work, pharmacy, dietetics, medicine (a consultant and a physician associate), nursing, occupational therapy and social work. There were a few unregistered assistants in nursing, occupational therapy and physiotherapy, and two allied health professional students.

### Course evaluation design

The study design was based on the Kirkpatrick model of evaluation [50]. While several theoretical models exist for the evaluation of professional training programmes, the model developed by Kirkpatrick has been used by training organisations for over 40 years, offering an established and comprehensive strategy. Key to the model is the premise that evaluation should go beyond the immediate reactions of attendees and should consider changed behaviours and professional practice. This is important as even when satisfaction ratings are good and learning objectives are met, transfer of knowledge into behaviour may not occur [51]. The model identifies the need for four levels of evaluation: level one explores participants’ initial reaction to training; level two identifies participants’ learning; level three investigates participants’ behaviour in applying what they have learnt from the training; and level four identifies the degree to which targeted results and outcomes are achieved. The study adopted a mixed methods, sequential approach using questionnaires and then individual interviews to collect data on each of the levels.

#### Data Collection

All participants were invited to complete a questionnaire prior to starting the course so that expectations and pre-course experience could be captured. An initial impact evaluation was then carried out whereby all participants were invited to immediately feedback about their experience through an evaluation questionnaire, which explored the first two levels of Kirkpatrick’s model: the participants’ initial reaction to the training and their learning. The questionnaire, with content area aligned with course aims and informed by the literature review, contained a series of closed questions using a Likert scale and three open questions with some free text comments invited. All participants (n = 49) who attended the course were invited to complete the questionnaires: 44 completed them pre-course and 47 post-course.

A further round of evaluation was carried out once participants had returned to practice and had a chance to apply their learning (2–5 months after completing the course). The aim was to explore the participants’ perceptions of how they had applied their learning from the course into their practice, any barriers encountered, and perceived outcomes, thus allowing the two further levels from Kirkpatrick’s model, behaviour and results, to be examined. Semi-structured interviews were conducted because of the opportunity they gave participants to explain in detail their experiences of applying learning from the course into practice with examples, thus providing rich data. An interview schedule was devised to ensure each of the study aims was explored. Questions related to: learning from the course; delivery of the course (simulation and interprofessional learning); application of learning in every day practice; examples of working differently; and perceived outcomes for patients/families. The semi structured nature of the interviews allowed further probing questions to be asked to elicit more information or for clarification [52]. All of the participants who attended the course were invited to be interviewed by email. An initial email was followed up with two subsequent emails and telephone contact. However, it was only possible to recruit 9 participants to the study. This was in part due to the length of time since the course had run and staff movement, as well as workload. The interviews were all conducted by one researcher, over the telephone and, with permission from participants, they were recorded and transcribed. The nine participants were from occupational therapy, nursing, physiotherapy and social work. One participant was community based while the rest were based in hospitals. Seven were registered professionals, of varied seniority, one was in an assistant practitioner role and another was a student.

#### Data analysis

The questionnaire data were manually entered into an Excel spreadsheet and descriptive statistics (frequencies and percentages) were calculated. The questionnaires’ open comments and the interview data were initially analysed by one researcher using thematic analysis [53,54], which involved six stages: familiarisation with the data; generating initial codes; searching for themes among codes; reviewing themes; defining and naming themes; producing a final report. A second researcher reviewed the initial analysis and together they refined the themes further. NVivo (a qualitative data analysis computer package) was used to assist with data management and coding.

#### Ethical issues

Ethical approval was obtained from a University Research Ethics Committee. As the study design met the UK’s Health Research Authority criteria of a service evaluation and did not directly involve patients, an NHS research ethics committee application was not required. Participation in the evaluation was voluntary. Questionnaires were completed anonymously and data were initially stored on an NHS secure computer then securely transferred to a university computer for analysis. Interview participants were provided with an information sheet and a consent form in advance and they gave verbal consent before the interview started. The interview data were anonymized and kept securely on a university password protected computer.

## Results

This section presents the results from the questionnaires and interviews.

### Questionnaire results

#### Pre course results

In the pre-course questionnaires, 30 (68%) of the 44 respondents reported they had experienced difficulty in transferring or receiving the care of an older patient with complex needs. Most comments about what would have helped them to manage the situation better related to communication issues: more direct communication between settings and other teams (hospital, community, care home), better communication within the multidisciplinary team (MDT), and improved communication with families. There were a number of comments about information quality and transfer of information: more information, greater accuracy of information, more timely information, more detailed handovers, higher quality referrals and being able to access baseline information about the patient or situation. Another area related to improved skills and more knowledge about the services and resources available within the community, the procedures for accessing these and referral processes in different London boroughs. Several respondents considered that support from more experienced colleagues would have helped and others identified better involvement of families.

#### Post course results

In the post-course questionnaires participants provided feedback that informed the first two levels of Kirkpatrick’s model of evaluation; initial reaction and learning [50]. The evaluation was focused on the course as a whole, rather than individual components or specific simulation strategies. The participants were asked to respond to four care transition-related closed questions using a Likert scale (see Table 2).

**Table 2:** Post-course questionnaires: participants’ perceptions.

	Totally disagree	Strongly disagree	Not sure	Agree	Strongly agree	Totally agree	Total

I recognise my role is vital in facilitating the safe transfer of patient care	0	0	1 (2.1%)	7 (14.9%)	9 (19.1%)	30 (63.8%)	47
I understand the relevance of effective communication and early information sharing.	0	0	0	5 (10.6%)	9 (19.1%)	33 (70.2%)	47
I am confident about involving service users and families in the discharge-planning and decision-making processes	0	0	0	11 (23.4%)	13 (27.7%)	23 (48.9%)	47
I am confident to assess and make decisions regarding a patient’s discharge needs and their discharge readiness	0	0	1 (2.1%)	13 (27.7%)	9 (19.1%)	22 (46.8%)	45

Participants were asked, through an open question, to identify up to three points that they had learnt during the course and a further open question asked for an example of learning that participants would take back to their clinical workplace following participation in the course. Table 3 provides a summary of the participants’ responses.

**Table 3:** Summary of participants’ open comments about their learning and intended actions.

Summary of participants’ learning from the course	Participants’ intended actions in their workplace

Increased empathy towards older people and the limitations and difficulties they may face during the discharge process; Greater understanding of the multidisciplinary team and the roles and difficulties faced by other professionals involved in the process of care transitions home; The importance of good interprofessional collaboration across the professions and the sharing of information; The factors that promote a successful care transition; The personal and communication skills needed for working with older people with complex needs.	More empathetic approach: establishing a relationship with the patient early on and being person-centred and sensitive to older people’s needs Increased involvement of patients and families in planning care transitions Improved communication and interprofessional collaboration across the care settings Ensure there is clarity about who is responsible for different roles and actions during care transitions and ensure that each health professional feels valued Be more proactive: anticipate problems and have back-up plans Educate colleagues about care transitions home e.g. ensure inclusion in junior staff induction Reflect on what has worked in care transitions and what could have been improved Apply their increased understanding of consent and mental capacity to care transitions Apply their increased awareness of local processes for care transitions and documentation

### Findings from interviews

The mode of delivery of the course – simulation – drew many positive comments during the interviews and seemed to enrich learning due to the opportunity to reflect and discuss practice that was true to life, supporting previous findings that realism of simulation scenarios impacts on learning [55]. The main themes were: Understanding individual needs and empathy; Communicating with patients and families; Interprofessional working; Working across settings to achieve effective care transitions. Findings within each theme relate to both level 3 and level 4 of Kirkpatrick’s model of evaluation [50] illustrating how participants applied what they had learnt and the degree to which results were achieved.

#### Understanding individual needs and empathy

The course provided insight into patients’ experience and in particular the simulation suit enabled them to experience what it was like to be older, which encouraged empathy:

‘For me, that was an eye opener because we sometimes take for granted that the older person is just as fit and can do all the things how we can do … that was scary – … and I thought ‘How must that feel for older people: to be completely and entirely dependent on us and physios and OTs?’ So that was one big thing for me that I took away from that day.’ (Participant 3)

As well as learning about physical constraints through the simulation activities, participants gained insights into emotional issues such as loss of independence and fear:

‘I found that incredibly insightful for me because a lot of the time, my role is [that] we put the care in place for people, but we don’t always think about what that [care package] means to that person when we put that care in place and how you are removing someone’s independence from them, and maybe not also fully understanding exactly what their needs are, and so it was really good.’ (Participant 8)

Participant 8 went on to describe how her learning from the course had directly influenced the support packages put in place for people when they go home, to ensure they really met the needs of the patients and she now actively tried:

‘To genuinely engage in the experience that these people have, to have an understanding of what the care is going to look like for this person.’ (Participant 8).

Some participants identified learning around the impact of the discharge process on the patient and family, particularly when it does not go smoothly:

‘I mean, for example, a discharge date being set and then cancelled at the very last minute, you know, the impact that that would have on not just the patient but the patient’s relatives, other staff, there’s a whole catalogue that goes on from a failed discharge.’ (Participant 2)

#### Communicating with patients and families

The staff reported that they had improved their communication with patients and families, to ensure a smooth transition home:

‘I think they felt a lot more in the loop and it helps with their anxiety, knowing what we’re doing as we go along and the process.’ (Participant 5)

One participant described how she now made contact with a patient’s next of kin before booking transport to ensure the next of kin knew and arrangements were made to ensure a smooth transition home. The participant said that she had not previously done that:

‘It’s maybe that the next-of-kin are not there or they don’t answer. So we make time in advance so instead of saying “I’ll do that tomorrow”, “I’ll do it today” or “at handover” we say to contact the next-of-kin before booking the transport.’ (Participant 9)

Other participants discussed how they now ensured that patients are involved in decision-making about discharge, that their view point is taken seriously and that family members are also fully informed:

‘To spend time, to spend that time rather than just dismissing it if the patient can’t communicate or they’re so deaf you can’t really communicate, well then, talk to the family […] it’s important to ensure that our patients understand what’s going on.’ (Participant 2)

‘To make them comfortable with the decision they’re making, that it is their decision and they’ve been involved in their care.’ (Participant 1)

#### Interprofessional working

All of those interviewed found that the interprofessional learning created a richer learning environment and offered insight into each other’s roles and ways of working:

‘I felt like they’d got a little bit more insight into what we do, we’re often not based on a ward, we don’t work for the NHS [National Health Service], but we are an integral part of the discharge planning, so for other people to get an understanding of what we do is really helpful.’ (Participant 8)

Participants now recognised that other professionals in the team could have different perspectives from their own:

‘Often, if you’re in one profession, you stick with that in your mind and you’re on a straight and narrow and that’s pretty much it, but if you’ve got other people coming in, a nurse will think about it differently, an OT, they all have a different way of approaching these kind of conversations and this kind of situation. So it’s quite helpful to know how they would do it.’ (Participant 1)

The course widened participants’ understanding of different professional roles that support patients across the whole care transition:

‘Primarily I deal with OTs, physios, doctors and nurses all the time so we’re quite well braced in how we engage with that aspect of the MDT, but not these other peripheral roles that we don’t always come into direct contact with, and again that’s helpful in understanding the journey of the patient and not just your part in that journey.’ (Participant 8).

This new understanding of other professional roles resulted in an increased appreciation of the challenges faced by others too:

‘But then I realised that their job is just as challenging as my job. So, although we are all in one team, each and everyone’s roles is just as important.’ (Participant 3).

Ensuring effective, structured communication within the multidisciplinary team while planning care transitions was considered an important learning point, arising from the course:

‘I think the key learning points were how important it is, communication within the MDT [multidisciplinary team].’ (Participant 5)

Participants identified changes in how they worked with other professionals, following the course, for example, working harder to communicate across the team and making better use of the range of services available in order to improve the patient journey:

‘An example of that, just asking people to start thinking about other ways that they might want to communicate and using other staff members, bringing in the speech and language therapist to help, to communicate more effectively with a patient, which may have been a bit more hit and miss before.’ (Participant 2)

Some participants also identified changes that were happening and had been driven more quickly as a result of the course:

‘We’re becoming more and more integrated anyway in the way that we’re working. Since that training, I can’t say it’s a direct impact, but certainly our team has been evolving and that training is part of that evolution to become more integrated within the MDTs.’ (Participant 8)

#### Working across settings to achieve effective care transitions

There were several examples of how participants were now working harder to communicate across the settings and make better use of the range of services available in order to improve the patient journey:

‘I always now, if the patient is on Warfarin, I liaise with the pharmacist before the discharge is done. Make sure we send the district nurse referral for him. And that was mainly because … it really hit me hard that there are these things happening, which I wasn’t aware of.’ (Participant 9)

These changes to practice were seen to benefit patients and families as they would experience a better co-ordinated and joined-up service that was more likely to result in a high quality transition home so that:

‘Service users or patients and their families aren’t feeling that they’re talking to lots of disparate unconnected groups of people where they’re having to repeat themselves or they’re feeling they’re getting inconsistent responses from different agencies who aren’t communicating effectively within themselves.’ (Participant 8)

Participants felt they had gained a much greater knowledge of the entire process for discharge home across settings and beyond their own role, and had learned what other resources were available that they could draw on:

‘But I now know what is available for these people, so I can help their way through it and things like that and they’re not going to be left on their own.’ (Participant 4)

Participants had gained insights into effective planning for care transitions and they discussed starting planning earlier than previously so that issues that could cause delays could be identified so: ‘We would have already tackled the things that would have delayed a discharge’ (Participant 6). Participants identified the importance of a holistic approach to discharge from hospital and not discharging too soon:

‘I took the holistic way of discharging because, in the wards we have had so many unsuccessful discharges and the way you all brought it out is to make sure the patient is ready to go and only then discharge the patient.’ (Participant 9)

## Discussion

This paper contributes by providing a detailed account of the development, implementatiion and evaluation of an interprofessional simulation course, to support integrated care initiatives to improve care transitions for older people with complex needs. As this was a pilot project, a limitation was the small sample size. However, the sample included participants from varied professions, from a range of seniority levels and from both hospital and community settings, which enabled an exploration of how inter-professional learning, and bringing hospital and community staff to learn together, impacted on integrated care in practice. An extremely high response rate was achieved for the pre and post intervention questionnaire (92% of participants completed both parts). While the sample size for the qualitative interviews was small, these were intended to be exploratory and provide in depth insight into participants’ experiences of learning and its application in practice. By the final interviews no new themes were emerging and saturation could be seen to have been achieved [56]. A further strength was the sequential approach which captured staff perceptions of their learning prior to attendance on the course, upon immediate completion and after they had applied their learning in practice, when planning care transfers for older people. The depth of the interview data meant that it was possible to gain an understanding of whether immediate knowledge gain translated into changed behaviour in practice. The evaluation relied on self reported data and there are limitations of this including social desirability bias and acquiescence bias [57]. However, self reported data in commonly used in evaluations and have been shown to be more accurate in measuring learning when used with samples who are used to reflecting on and assessing their own learning, such as this population group of health and social care professionals [57]. In order to reduce potential bias in this area non leading questions were used in the interview schedule and participants were asked to provide examples from practice to support their reflections. A further limitation was that the design did not include data collection with older people and their families nor measure of impact on outcomes such as reduced readmission rates, but this was outside the scope of this pilot project.

Goodwin et al. [7] suggested that, to achieve integrated care, ‘what appears to matter most is not the organisational solution but what happens at the service- and clinical-level’. Therefore, the health and social care professionals who deliver integrated care in clinical practice are of central importance yet, integrated care initiatives have not always recognized and addressed the associated educational needs of the health and social care workforce [8]. The starting point for the current project was an identified need to educate healthcare professionals who were involved in care transitions from hospital to home for older people with complex needs, in a virtual integrated care system. That the scoping exercise that informed the course development identified a preference for interprofessional education, indicated workforce recognition of the importance of effective interprofessional working. Furthermore, the pre course questionnaire results revealed that difficulties encountered when managing care transitions to home often related to collaborative working: communication difficulties across professions and lack of knowledge of services and resources in other settings. These results support previous studies that have highlighted deficits in communication and information transfer during care transitions from hospital [58,59].

The findings from the current study indicated that a simulation course where health and social care professionals from across hospital and community settings learned interprofessionally, was perceived to lead to a more collaborative and integrated way of working in practice. The care of older people increasingly needs a more interprofessional collaborative approach to deliver the necessary complex and continuous care and overall, effects of interprofessional interventions for older people have been identified as being positive on a number of outcomes, including care transitions [60]. Internationally, there is growing interest in the ability of healthcare professionals to work collaboratively together [61] with collaborative practice being considered vital for providing safe, high quality, patient-centred care [41]. Collaboration is a complex process that presents many challenges [62] but it is increasingly understood as an interpersonal process that requires trust, mutual respect and effective communication [19,63], with regular dialogue between the professionals involved [64,15]. Similarly, for successful integrated care, there is a need to create trust and mutual respect between professionals [8] and to recognise the importance of issues such as relationship building and fostering an environment that supports new collaborations and ways of working [7].

Healthcare students have been found to be positive towards inter-professional collaboration and learning [65] but many health and social care professionals have not had interprofessional learning opportunities [8]. The findings from both the post course questionnaire and the follow up interviews showed that the interprofessional simulation during the course was highly valued by participants, that it contributed to a richer learning environment and successfully promoted collaborative practice between the participating health and social care professionals. Whilst bringing professionals together for education is challenging, it can have a positive effect on outcomes [35]. In the current study, involving health and social care professionals in the scoping exercise in the planning of the course appeared to positively affect their willingness to participate in the course themselves or to support other staff in attending.

Howarth et al. [66] suggested that for successful integrated care, there needs to be role awareness and effective communication between professional groups within teams. However, practitioners in different settings often work independently, with little knowledge of other settings [12,24,13]. Staff have often not worked within the settings to which they are transferring patients and so they may be unfamiliar with their services [67]. Previous research findings revealed that community and acute hospital staff can lack opportunities to meet each other, build relationships, develop trust and gain understanding of each other’s roles and the service provision in other parts of the system [68,15]. The benefits of facilitating a regular dialogue between team members are well recognised [69,64] and previous research findings highlighted that strategies to bring professionals together to learn about each other’s services could be successful [63]. In the current study, the course acted as a catalyst to bring health and social care professionals from across settings together, which resulted in a greater understanding of the roles and difficulties encountered by other professionals across the hospital-community interface in the process of care transfers home and the importance of sharing information, communication and effective interprofessional team work. This learning translated into practice as at interview, participants were able to identify examples of how they had improved interprofessional communication and strategies for working across settings to achieve more effective care transitions home for older people.

Providing effective interprofessional education can, however, present some challenges as learners from different professions may have different ways of interacting with the world, use different professional languages and have different preferred learning styles [41,70]. Underpinned by Kolb’s [71] premise that people learn best by doing, reflecting and making modifications to their practice, simulation provides the catalyst for learning, through which there is an opportunity for interprofessional education to occur, with knowledge created in the social exchange among participants. The situated learning approach, which sees learning as a social process whereby knowledge is co-constructed by participants and is informed by its context, invites integration into a community of practice, which fosters interaction and encourages sharing of ideas [72], This approach also encourages individual and group analysis of the activity systems in which they operate [73] so shared understandings may be constructed. Interprofessional simulation is has been found to offer a learning environment that supports acquisition of the knowledge, skills, attitudes, and behaviours of teamwork required to promote safe quality patient care [41]. This study supports these conclusions about the use of simulation, with participants expressing appreciation for this mode of delivery, seeing it as having provided an enriched learning environment, which contributed to the learning outcomes and subsequent changes in ways of working. An authentic simulation experience has been identified as important for optimizing learning [74] and in the current study, the opportunity to experience simulated practice that was true to life upon which participants could reflect and discuss was an effective learning strategy. It should be acknowledged however that simulation is a resource intensive educational approach with associated cost implications [75].

The way older people are treated by staff has been found to have a major impact on their overall care experiences [5]. In the current study, findings indicated that simulation directly contributed to participants’ perceptions of increased empathy and understanding of the physical and emotional needs of older people with complex needs. Whilst many studies that have evaluated simulation have been based in acute care, Alcorn et at. [76] found that a simulation course improved medical students’ perceived ability to care for older people. The level of patient involvement in care transition processes is important for successful transitional care [9,12] but internationally, previous studies have highlighted that older people may not be as involved in decision making about care transitions as they would prefer [77,15,16] and that poor communication with patients adversely affects transition experiences [13,5,10,78,79]. In the current study, participants discussed that, as a result of the course, they now better understood the need to communicate more effectively with patients and their families and they gave examples of how they now involved them more in decision making about their care transfers.

## Conclusion

Delivering integrated care in practice requires a health and social care workforce that can work interprofessionally and collaboratively in a person-centred way. However, the workforce’s educational needs for delivering effective integrated care have not always been fully acknowledged and addressed. This paper reported on how an educational intervention to support integrated care for older people experiencing care transitions from hospital to home could be planned and delivered. The simulation approach and interprofessional nature of the course was well evaluated and contributed to improved empathy with older people and a better understanding of other professional roles and collaborative practice. The key areas of learning that were identified during the immediate post-course evaluation, including better patient and family involvement, were retained after participants returned to practice. The evaluation also indicated areas where, from participants’ perceptions, they applied their learning and changed their practice as a result of the course. The course was delivered as a pilot and so the small scale nature of the evaluation is a limitation. It is recommended that a larger scale evaluation, using a wider range of methods and data sources, and with measurement of benefits, could be conducted in the future.
